# Rapid Acting Insulin Use and Persistence among Elderly Type 2 Diabetes Patients Adding RAI to Oral Antidiabetes Drug Regimens

**DOI:** 10.1155/2016/5374931

**Published:** 2016-09-28

**Authors:** Usha Sambamoorthi, Arijita Deb, Steve Zhou, Rahul Garg, Tao Fan, Anders Boss

**Affiliations:** ^1^School of Pharmacy, West Virginia University, Morgantown, WV, USA; ^2^Sanofi US, Inc., Bridgewater, NJ, USA

## Abstract

We examined the real-world utilization and persistence of rapid acting insulin (RAI) in elderly patients with type 2 diabetes who added RAI to their drug (OAD) regimen. Insulin-naïve patients aged ≥65 years, with ≥1 OAD prescription during the baseline period, who were continuously enrolled in the US Humana Medicare Advantage insurance plan for 18 months and initiated RAI were included. Among patients with ≥2 RAI prescriptions (RAIp), persistence during the 12-month follow-up was assessed. Multivariate logistic regression analyses identified factors affecting RAI use and persistence. Of 3734 patients adding RAI to their OAD regimen, 2334 (62.5%) had a RAIp during follow-up. Factors associated with RAIp included using ≤2 OADs; cognitive impairment, basal insulin use during follow-up; and higher RAI out-of-pocket costs ($36 to <$56 versus $0 to $6.30). Patients were less likely to persist with RAI when on ≤2 OADs versus ≥3 OADs and when having higher RAI out-of-pocket costs ($36 to <$56 versus $0 to $6.30) and more likely to persist when they had cognitive impairment and basal insulin use during follow-up. Real-world persistence of RAI in insulin-naïve elderly patients with type 2 diabetes was very poor when RAI was added to an OAD regimen.

## 1. Introduction

Rapid acting insulins (RAIs) are characterized by fast-onset (10–15 minutes) and short duration of action (3–5 hours) and are used to manage postprandial glucose excursions in patients with type 2 diabetes [1, 2]. Current evidence-based clinical practice guidelines recommend the use of RAI in combination with basal insulin when postprandial glycemic goals are not met with a regimen consisting of basal insulin and oral anti-diabetic drugs (OADs) [3]. However, with the increasing shift towards personalized treatment for the management of type 2 diabetes [3, 4], clinicians might resort to approaches that are not suggested in the current guidelines [5]. One such approach is to add RAI to an OAD regimen rather than following the standard practice of adding RAI to basal insulin in order to meet the needs of individual patients. This approach has been shown to be effective in clinical studies [6–8], in which the addition of RAI to an OAD regimen achieved targeted glycemic controls in type 2 diabetes patients. Most of these clinical studies used RAI in combination with metformin [6, 7].

Given the need to provide evidence-based personalized treatment, it is important to understand the extent to which RAI is added to OAD regimens and the characteristics of patients who are initiated on RAI and their persistence with this treatment. In addition, as the clinical effectiveness of RAI is tied to persistence, it is critical to understand the factors associated with persistence of RAI when RAI is added to an OAD regimen. A previous study in patients adding RAI to a basal insulin regimen reported poor persistence with RAI [9]. However, the pattern of persistence among type 2 diabetes patients adding RAI to their OAD regimen is unknown.

In the United States, the latest data from the Centers for Disease Control and Prevention suggest that over 25% of the adult population with diabetes is ≥65 years of age [10]. Furthermore, the Medicare-eligible population with diabetes is expected to increase in the coming years [11, 12]. Therefore, we conducted this study to describe the characteristics of elderly (≥65 years of age) Medicare beneficiaries with type 2 diabetes who added RAI to their OAD regimen and to describe the factors associated with RAI continuation and persistence in this patient group.

## 2. Methods

### 2.1. Study Design

This was an observational, retrospective cohort study using medical, pharmacy, and laboratory claims from US Humana insurance plans for the period 2007–2012. Humana is an integrated claims database and includes enrollment, medical, and pharmacy claims information for more than 12 million Humana members for both commercial and Medicare advantage plans across the US. For this study, we have only included patients who were enrolled in the Humana Medicare Advantage Prescription Drug plans. The first observed RAI prescription (RAI index date) was used to define the baseline and follow-up periods. The baseline period was 6 months prior to the RAI index date, and the follow-up period was 12 months after the RAI index date.

The study population comprised Medicare beneficiaries with type 2 diabetes who were ≥65 years of age at baseline and had 18-month continuous enrollment (6-month baseline and 12-month follow-up) in Medicare Advantage Prescription Drug (MAPD) plans, had ≥1 OAD claim during the baseline period, and had newly added RAI to their OAD regimen between July 2007 and December 2011. Diagnosis of type 2 diabetes was ascertained using claims for ≥1 inpatient visit or ≥2 physician visits at least 30 days apart and a primary or secondary diagnosis of type 2 diabetes using* International Classification of Diseases, 9th Revision, Clinical Modification* (ICD-9-CM) codes 250.x0 or 250.x2. Patients were excluded if they used any insulin during the baseline period and were enrolled only in commercial insurance plans or if their gender or insurance plan type was unknown.

### 2.2. Measures

RAI continuation was measured based on the number of RAI prescriptions during the 12-month follow-up period. Those with >1 RAI prescription during follow-up were considered to have continued with RAI.

Persistence with RAI was examined only in elderly Medicare beneficiaries who had ≥2 RAI prescriptions in the 12-month follow-up period using two measures of persistence based on published methods [9]. Persistence Measure 1 was defined as the absence of any 90-day gap between RAI prescriptions; that is, patients were considered persistent at 12 months if they did not have a 90-day gap by the end of the time period. In persistence Measure 2, persistence at 12 months was defined as having ≥4 RAI prescriptions in the 12-month follow-up period, with ≥1 RAI prescription in each quarter of the 12-month period. For persistence Measure 2, the counts of RAI prescriptions included the index RAI prescription.

Independent variables recorded at baseline included the following demographic and clinical characteristics: age, gender, race/ethnicity, region, and insurance plan type (health maintenance organizations [HMOs]; preferred provider organizations (PPOs); and private fee-for-service, [PFFS]/others), any hypoglycemia (identified using ICD-9-CM codes 250.8, 251.0, 251.1, and 251.2 for any emergency department [ED], inpatient, or outpatient visits [13]), and severe hypoglycemia (identified using ICD-9-CM codes 250.8, 251.0, 251.1, and 251.2 for inpatient or ED visits [13]), baseline glycosylated hemoglobin (HbA1c) value, diabetes complications using the adapted-Diabetes Complications Severity Index (aDCSI) score [14], healthcare utilization (any inpatient and any ED visit), and medication use (OADs and other concomitant medications). In addition, the following patient complexities specific to the elderly [15] were evaluated: cognitive impairment, depression, injurious falls (ICD-9-CM E-codes [16], and V-codes [17]), urinary incontinence (ICD-9-CM diagnosis codes), and polypharmacy [18]. Polypharmacy was based on the number of different therapeutic drug classes in the 90 days before index period. The definition is based on previous published literature [19]. It was defined as 1 standard deviation (SD) above the mean. Based on our data, those with more than 14 different therapeutic drug classes (mean = 10, SD = 4) during the 90-day preindex period were considered as having polypharmacy.

The independent variables from the 12-month follow-up period included average RAI out-of-pocket costs (quartiles: $0 to <$6.30; $6.30 to <$36; $36 to <$56; and ≥$56) and basal insulin use. The RAI out-of-pocket cost was measured as total out-of-pocket cost for RAI prescriptions divided by the total number of RAI prescriptions.

### 2.3. Statistical Analysis

Chi-square tests were used to assess the unadjusted subgroup differences in RAI continuation and RAI persistence. Multivariable logistic regressions were used for adjusted analyses. Factors used in regression models included number of OADs at baseline, gender, age, race, region, type of insurance plan, any hypoglycemic event at baseline, aDCSI score, HbA1c value at baseline, any inpatient visit, any ED visit, polypharmacy, cognitive impairment, depression, injurious fall, urinary incontinence, basal insulin use at follow-up, and average RAI out-of-pocket costs at follow-up. All analyses were conducted using SAS 9.3 (SAS Institute Inc., Cary, NC, USA).

## 3. Results

### 3.1. Study Population

A total of 16,850 Medicare beneficiaries ≥65 years of age with type 2 diabetes were identified as receiving RAI therapy between July 2007 and December 2011. Among these patients, 3734 (22.2%) added RAI to their OAD regimen and were included in this study. The baseline demographic and clinical characteristics of these 3734 patients are presented in Table 1. The mean age of this population was 72.9 years, 51% were female, nearly 80% were white, and 60% lived in US region South. During the baseline period, 46.2% were taking 1 OAD, 40.3% were taking 2 OADs, and 13.6% were taking ≥3 OADs. Further, 66% had an inpatient visit, 60% had an ED visit, and 14.4% had polypharmacy.

### 3.2. Basal Insulin Use and RAI Out-of-Pocket Costs over the 12-Month Follow-Up Period

Over the 12-month follow-up period, 52.8% of the patients who added RAI to their OAD regimen also added basal insulin. Approximately 25% of patients had RAI OOP cost in each of the four cost quartiles ($0 to <$6.30; $6.30 to <$36; $36 to <$56; and ≥$56).

### 3.3. Continuation of RAI Therapy at the 12-Month Follow-Up

The mean (standard deviation [SD]) number of RAI prescriptions was 3.5 (3.1) at the 12-month follow-up. Of the 3734 patients in the study population, 37.5% (*n* = 1400) had only 1 RAI prescription (i.e., discontinued RAI therapy), and 62.5% (*n* = 2334) had >1 RAI prescription (i.e., continued RAI therapy). Subgroup differences were observed between those who had only 1 RAI prescription and those who had ≥1 RAI prescription (continuation) (Table 2).

Multivariable logistic regression analysis showed that continuation of RAI at 12 months (i.e., ≥2 RAI prescriptions at 12 months) was significantly less likely in patients who were taking 1 OAD at baseline compared with those taking ≥3 OADs; were older (aged 70–79 years versus 65–69 years); lived in US region South versus the Northeast/West/other regions; had no diabetes-related complications; had any inpatient visit; and had higher out-of-pocket RAI costs (Table 3).

Continuation with RAI at 12 months was significantly more likely in patients with baseline A1C values 8.0–9.0% and >9.0% compared with HbA1c <8.0%, cognitive impairment, and depression and in those who added basal insulin during the 12-month follow-up period (Table 3).

### 3.4. RAI Persistence

There were significant subgroup differences in the percentage of patients who persisted and did not persist with RAI therapy at 12 months as demonstrated using bivariate analysis (Table 4).

Based on multivariable logistic regression analysis, the baseline factors found to be significantly associated with RAI persistence (Measure 1) at 12 months were cognitive impairment and addition of basal insulin during follow-up. Those patients taking 1 or 2 OADs versus ≥3 OADs at baseline and those having higher OOP costs for RAI prescriptions were significantly less likely to persist (Table 5). Similar findings were observed with RAI persistence Measure 2 (filling ≥1 RAI prescription every 3 months). Additionally, persistence Measure 2 demonstrated that individuals living in US region South compared with Northeast/West/other regions, and patients with polypharmacy were less likely to persist with RAI (Table 5).

### 3.5. Sensitivity Analysis

We conducted a sensitivity analysis using an alternative measure of out-of-pocket costs over the 12-month follow-up period. For each person, it was calculated as total out-of-pocket cost for RAI prescriptions divided by the total number of days supplied for RAI prescriptions. This was multiplied by 30 in order to interpret the out-of-pocket cost per 30-day supply. The RAI out-of-pocket cost quartile was as follows: quartiles: $0 to <$6.4; $6.4 to <$35.5; $35.5 to <$47.6; and ≥$47.6.

Results from both bivariate (Tables 2 and 3) and multivariate analyses using the alternate definition of RAI out-of-pocket suggest that the direction and magnitude of the associations between RAI out-of-pocket costs and RAI continuation and RAI persistence did not change. For example, compared to patients with RAI out-of-pocket cost per 30-day supply ranging between $0 and <$6.4, those with RAI out-of-pocket cost ranging between $6.4 and <$35.5 (AOR = 0.43, 95% CI = 0.31, 0.59) were significantly less likely to be persistent (not shown in Tabular form).

## 4. Discussion

This is the first real-world study using data from an administrative claims database to examine the practice-based addition of RAI to an OAD regimen in elderly patients with type 2 diabetes. Although intensification of OAD therapy with RAI is not a standard of care for postprandial glycemic management in type 2 diabetes, clinical studies have shown the benefits of such practice [6, 7]. Here, we report for the first time practice-based evidence showing that a sizeable proportion of elderly type 2 diabetes patients added RAI therapy to their OAD regimen.

Management of type 2 diabetes patients by treatment intensification of OADs with RAI may be considered in two groups of patients, those in care homes that use RAI as a supplemental dose temporarily and those that use RAI on a regular basis. Clinicians may be uncertain regarding the optimal strategy for insulin intensification [20] or favor resorting to less widely used treatment approaches to meet the needs of individual patients [5], as personalized diabetes management is becoming important. In addition, for patients with HbA1c <8.4%, the postprandial contribution to hyperglycemia is more important than the basal contribution, and therefore, targeting postprandial hyperglycemia with RAI is a relevant option [21]. However, this can result in increased weight gain and hypoglycemia, which is associated with all insulin therapy use but is more pronounced when treatment is intensified with RAI.

A limited number of studies have demonstrated the benefits of adding RAI to an OAD regimen for achieving targeted glycemic control in type 2 diabetes patients [6–8]. The present study did not explore the effect of this approach on HbA1c levels because baseline and follow-up HbA1c data were not available for all patients.

Having initiated RAI therapy, RAI use was found to be temporary for many of the patients in our study. We report a persistence rate of 18.4% with persistence Measure 1 in this study. This low persistence rate is similar to that reported in a study that used the same measure to evaluate RAI persistence in an adult type 2 diabetes population when adding RAI to a basal insulin regimen (19.1%) [9].

There was a significant difference between the two measures of persistence, Measure 1 and Measure 2 (18.4% versus 33.7%, resp.). The difference between the two measures was not surprising as persistence Measure 1—with a 90-day gap—was more conservative than Measure 2, which had ≥1 prescription per quarter. As physicians often titrate RAI dose, a 90-day gap might not necessarily mean discontinuation of therapy. So a more lenient measure of persistence (Measure 2) is often used.

We identified that baseline use of 1 or 2 OADs versus ≥3 OADs was significantly associated with RAI nonpersistence. Use of fewer OADs at baseline could indicate that the diabetes stage was not very severe in patients, whereas use of ≥3 OADs at baseline may indicate more severe or uncontrolled diabetes that would require treatment intensification with RAI. Patients with high out-of-pocket costs were also significantly less likely to have RAI persistence over a 12-month period. This is in line with other studies that have also reported high out-of-pocket costs for insulin prescriptions in general [22–24] and for RAI specifically, to be a barrier to RAI persistence [9]. It has also been suggested that insurance policies that eliminate copays or that lower copays may encourage RAI persistence [22]. Higher RAI out-of-pocket costs with lower RAI continuation and persistence were robust regardless of measures of out-of-pocket costs. These findings have implications for designing value-based insurance to eliminate copays or lower copays in order to encourage RAI persistence.

Interestingly, cognitive impairment is another factor we found to be associated with RAI persistence despite the expectation that this might adversely affect persistence to a drug regimen. It is possible that a patient's cognitive impairment may have been recognized by a family member and/or a healthcare provider, who could have taken steps to ensure that the insulin was administered under supervision, leading to greater persistence among the patients with cognitive impairment. It is plausible that these patients were institutionalized and therefore had caregivers. Further research may elucidate the reasons for greater persistence among the patients with cognitive impairment in our study population.

The augmentation of the RAI plus OAD regimen with basal insulin during the 12-month follow-up was another factor associated with RAI persistence. Basal insulin was initiated by 52.8% of patients in this study. It is plausible that these patients have needed further intensification of their insulin regimen.

Taken together, these findings suggest that therapies other than RAI plus OADs may be needed for personalized treatment and optimal diabetes care. Further research is required to understand the rationale for adding RAI to OAD regimens in elderly patients with type 2 diabetes, identify the unmet needs of these patients, and assess the comparative effectiveness of different treatment regimens used for personalizing therapy. Future research should also focus on the impact of RAI persistence on clinical outcomes (change in A1C outcomes) and economic outcomes such as diabetes realated healthcare cost and resource utilization.

*Limitations*. Several limitations of this study should be acknowledged. Coding errors (e.g., under- or overcoding) are a risk when using ICD-9-CM diagnosis codes in medical claims to identify type 2 diabetes, hypoglycemia, and diabetes complications. In addition, because of the methodology used in this study, minor hypoglycemia events may not have been detected. The nature of claims data means that prescriptions rather than actual use of medications were recorded, and RAI prescriptions may not have been added to claims databases. Moreover, the 90-day prescription gap measure of persistence may have underestimated the level of RAI persistence. Some data were unavailable from the database; thus, time from type 2 diabetes diagnosis to RAI initiation could not be controlled as type 2 diabetes diagnosis dates were unavailable, baseline HbA1c data were not available for the majority of patients, and clinical reasons for RAI initiation could not be examined in this study. Finally, the study included elderly Medicare beneficiaries enrolled in Humana MAPD plans, so the results may not be generalizable to all elderly Medicare beneficiaries with type 2 diabetes.

## 5. Conclusions

A sizeable proportion of elderly patients with type 2 diabetes have RAI added to their OAD regimen. However, use of RAI was temporary, and type 2 diabetes patients' persistence with RAI was very poor. Nonpersistence was associated with use of fewer OADs and higher out-of-pocket costs at baseline, suggesting that patients with less severe diabetes symptoms and those who had to pay extra for their prescription did not persist and that targeting these patient populations through patient education and insurance policies that eliminate copays or lower copays may encourage RAI persistence. Many patients augmented their RAI plus OAD regimen with basal insulin, and this, together with cognitive impairment, was associated with persistence of RAI. Further research to understand the rationale for addition of RAI to OAD among elderly patients with type 2 diabetes is warranted.

## Figures and Tables

**Table 1:** Characteristics of elderly (≥65 years) Medicare beneficiaries with type 2 diabetes mellitus who newly added rapid acting insulin to their oral antidiabetic drug regimen. Humana Medicare Advantage Prescription Drug Plan database 2007–2012.

	*N*	Column%
*All*	*3,734*	*100*
*Number of OADs*		
1 OAD	1,724	46.2
2 OADs	1,503	40.3
≥3 OADs	507	13.6

Demographic characteristics		

*Gender*		
Female	1,899	50.9
Male	1,835	49.1
*Race*		
White	2,971	79.6
African American	516	13.8
Others	157	4.2
Missing	90	2.41
*Age in years*		
65–69 years	1,296	34.7
70–74 years	1,090	29.2
75–79 years	764	20.5
80+ years	584	15.6
*Region*		
Midwest	1,122	30
South	2,227	59.6
Northeast/west/others	385	10.3

Health insurance characteristics		

*Type of plan*		
PFFS	1,311	35.1
HMO	1,435	38.4
PPO/others	988	26.5

Baseline clinical characteristics		

*Any hypoglycemia*		
Yes	469	12.6
No	3,265	87.4
*Severe hypoglycemia*		
Yes	365	9.8
No	3,369	90.2
*aDCSI score*		
0	926	24.8
1-2	1,141	30.6
3-4	595	15.9
5–13	1,072	28.7
*A1C*		
<7%	377	10.1
7-8%	298	8
8-9%	234	6.3
>9%	332	8.9
Not available	2,493	66.8

Baseline healthcare utilization		

*Any inpatient visit*		
Yes	2,460	65.9
No	1,274	34.1
*Any ER visit*		
Yes	2,229	59.7
No	1,505	40.3

Special conditions for the elderly		

*Polypharmacy*		
Yes (>14)	537	14.4
No (≤14)	3,197	85.6
*Cognitive impairment*		
Yes	677	18.1
No	3,057	81.9
*Depression*		
Yes	608	16.3
No	3,126	83.7
*Fall*		
Yes	236	6.3
No	3,498	93.7
*Urinary incontinence*		
Yes	173	4.6
No	3,561	95.4

One-year follow-up basal insulin use		

*Basal insulin*		
Yes	1,973	52.8
No	1,761	47.2

One-year follow-up RAI out-of-pocket cost per RAI prescription		

*Average cost per RAI prescription*		
$0.0–<$6.3	933	25
$6.3–<$36	945	25.3
$36–<$56	923	24.7
≥$56	933	25

One-year follow-up RAI out-of-pocket cost per 30-day supply		

*RAI OOP cost per 30-day supply*		
$0.0–<$6.4	934	25
$6.4–<$35.5	933	25
$35.5–<$47.6	934	25
≥$47.6	933	25

Note: based on 3,734 elderly Medicare beneficiaries aged 65 years and above with type 2 diabetes mellitus, who were continuously enrolled in the Humana Medicare prescription drug plans for 18 months between 2007 and 2011 and added rapid acting insulin to their oral antidiabetic drugs regimen.

A1C: glycated hemoglobin; aDCSI: adapted-Diabetes Complications Severity Index; ER: emergency room; HMO: health maintenance organization; OAD: oral antidiabetic drug; PFFS: private fee-for-service; PPO: preferred provider organization; RAI: rapid acting insulin.

**Table 2:** Description of elderly (≥65 years) Medicare beneficiaries with type 2 diabetes mellitus who newly added rapid acting insulin to their oral antidiabetic drug regimen. Single versus multiple rapid acting insulin prescription during one-year follow-up. Humana Medicare Advantage Prescription Drug Plan database 2007–2012.

	Single RAI		Multiple RAI		*P* value
	*N*	Row%	*N*	Row%	
*All (N* = 3,734)	*1,400*	*37.5*	*2,334*	*62.5*	
*Number of OADs*					<0.001
1 OAD	720	41.8	1,004	58.2	
2 OADs	526	35.0	977	65.0	
≥3 OADs	154	30.4	353	69.6	

Demographic characteristics					

*Gender*					
Female	693	36.5	1,206	63.5	
Male	707	38.5	1,128	61.5	
*Race*					
White	1,117	37.6	1,854	62.4	
African American	203	39.3	313	60.7	
Others	56	35.7	101	64.3	
Missing	24	26.7	66	73.3	
*Age in years*					<0.001
65–69 years	436	33.6	860	66.4	
70–74 years	411	37.7	679	62.3	
75–79 years	331	43.3	433	56.7	
80+ years	222	38.0	362	62.0	
*Region*					<0.001
Midwest	354	31.6	768	68.4	
South	920	41.3	1,307	58.7	
Northeast/west/others	126	32.7	259	67.3	

Health insurance characteristics					

*Type of plan*					
PFFS	479	36.5	832	63.5	
HMO	564	39.3	871	60.7	
PPO/others	357	36.1	631	63.9	

Baseline clinical characteristics					

*Any hypoglycemia*					<0.05
Yes	201	42.9	268	57.1	
No	1,199	36.7	2,066	63.3	
*Severe hypoglycemia*					<0.05
Yes	159	43.6	206	56.4	
No	1,241	36.8	2,128	63.2	
*aDCSI score*					
0	326	35.2	600	64.8	
1-2	424	37.2	717	62.8	
3-4	241	40.5	354	59.5	
5–13	409	38.2	663	61.8	
*A1C*					<0.001
<8%	303	44.9	372	55.1	
8-9%	71	30.3	163	69.7	
>9%	82	24.7	250	75.3	
Not available	944	37.9	1,549	62.1	

Baseline healthcare utilization					

*Any inpatient visit*					<0.001
Yes	1,032	42.0	1,428	58.0	
No	368	28.9	906	71.1	
*Any ER visit*					<0.001
Yes	911	40.9	1,318	59.1	
No	489	32.5	1,016	67.5	

Special conditions for the elderly					

*Polypharmacy*					<0.05
Yes (>14)	228	42.5	309	57.5	
No (≤14)	1,172	36.7	2,025	63.3	
*Cognitive impairment*					<0.01
Yes	224	33.1	453	66.9	
No	1,176	38.5	1,881	61.5	
*Depression*					
Yes	211	34.7	397	65.3	
No	1,189	38.0	1,937	62.0	
*Fall*					
Yes	96	40.7	140	59.3	
No	1,304	37.3	2,194	62.7	
*Urinary incontinence*					
Yes	63	36.4	110	63.6	
No	1,337	37.5	2,224	62.5	

One-year follow-up basal insulin use					

*Basal insulin*					<0.001
Yes	553	28.0	1,420	72.0	
No	847	48.1	914	51.9	

One-year follow-up RAI out-of-pocket cost per RAI prescription					

*Average cost per RAI prescription*					<0.001
$0.0–<$6.3	308	33.0	625	67.0	
$6.3–<$36	420	44.9	516	55.1	
$36–<$56	425	45.6	507	54.4	
≥$56	247	26.5	686	73.5	

One-year follow-up RAI out-of-pocket cost per 30-day supply					

*RAI OOP cost per 30-day supply*					<0.001
$0.0–<$6.4	283	30.3	651	69.7	
$6.4–<$35.5	409	43.8	524	56.2	
$35.5–<$47.6	403	43.1	531	56.9	
≥$47.6	305	32.7	628	67.3	

Note: based on 3,734 elderly Medicare beneficiaries aged 65 years and above with type 2 diabetes mellitus, who were continuously enrolled in the Humana Medicare prescription drug plans for 18 months between 2007 and 2011 and added rapid acting insulin to their oral antidiabetic drugs regimen. Significant group differences by multiple versus single prescription for rapid acting insulin were tested using chi-square tests.

A1C: glycated hemoglobin; aDCSI: adapted-Diabetes Complications Severity Index; ER: emergency room; HMO: health maintenance organization; OAD: oral antidiabetic drug; PFFS: private fee-for-service; PPO: preferred provider organization; RAI: rapid acting insulin.

**Table 3:** Adjusted odds ratios and 95% confidence intervals from logistic regressions of multiple versus single rapid acting insulin prescriptions during 12-month follow-up among elderly (≥65 years) Medicare beneficiaries with type 2 diabetes mellitus who added rapid acting insulin to their oral antidiabetic drug. Humana Medicare Advantage Prescription Drug Plan database 2007–2012.

	AOR	95% CI	*P* value
*Number of OADs*			
1 OAD	0.72	[0.58, 0.91]	<0.01
2 OADs	0.92	[0.73, 1.16]	
≥3 OADs			

Demographic characteristics			

*Gender*			
Female	1.11	[0.96, 1.29]	
Male			
*Race*			
White			
African American	0.98	[0.80, 1.22]	
Others	0.92	[0.64, 1.32]	
Missing	1.64	[0.99, 2.72]	
*Age in years*			
65–69 years			
70–74 years	0.83	[0.70, 1.00]	<0.05
75–79 years	0.71	[0.58, 0.87]	<0.001
80+ years	0.96	[0.76, 1.21]	
*Region*			
Midwest	1.05	[0.80, 1.36]	
South	0.68	[0.53, 0.88]	<0.01
Northeast/west/others			

Health insurance characteristics			

*Type of plan*			
PFFS			
HMO	1.01	[0.84, 1.22]	
PPO/others	0.98	[0.82, 1.18]	

Baseline clinical characteristics			

*Any hypoglycemia*			
Yes	0.82	[0.66, 1.01]	
No			
*aDCSI score*			
0	0.82	[0.66, 1.02]	<0.01
1-2	0.88	[0.73, 1.07]	
3-4	0.82	[0.66, 1.02]	
5–13			
*A1C*			
<8%			
8-9%	1.48	[1.05, 2.07]	<0.05
>9%	1.94	[1.42, 2.65]	<0.001
Not available	1.21	[0.99, 1.47]	

Baseline healthcare utilization			

*Any inpatient visit*			
Yes	0.54	[0.44, 0.65]	<0.001
No			
*Any ER visit*			
Yes	0.90	[0.75, 1.07]	
No			

Special conditions for the elderly			

*Polypharmacy*			
Yes (>14)	0.88	[0.72, 1.09]	
No (≤14)			
*Cognitive impairment*			
Yes	1.51	[1.23, 1.85]	<0.001
No			
*Depression*			
Yes	1.23	[1.00, 1.51]	<0.05
No			
*Fall*			
Yes	0.88	[0.65, 1.18]	
No			
*Urinary incontinence*			
Yes	1.08	[0.76, 1.52]	
No			

One-year follow-up basal insulin use			

*Basal insulin*			
Yes	2.30	[1.99, 2.66]	<0.001
No			

One-year follow-up RAI out-of-pocket cost per RAI prescription			

*Average OOP cost per RAI prescription*			
$0.0–<$6.3			
$6.3–<$36	0.55	[0.45, 0.67]	<0.001
$36–<$56	0.53	[0.43, 0.65]	<0.001
≥$56	1.10	[0.89, 1.38]	

Note: based on 3,734 elderly Medicare beneficiaries aged 65 years and above with type 2 diabetes mellitus, who were continuously enrolled in the Humana Medicare prescription drug plans for 18 months between 2007 and 2011 and added rapid acting insulin to their oral antidiabetic drugs regimen. The reference group for the dependent variable is having more than one RAI prescription during the one-year follow-up period.

A1C: glycated hemoglobin; aDCSI: adapted-Diabetes Complications Severity Index; AOR: adjusted odds ratio; CI: confidence interval; ER: emergency room; HMO: health maintenance organization; OAD: oral antidiabetic drug; PFFS: private fee-for-service; PPO: preferred provider organization; RAI: rapid acting insulin.

**Table 4:** Description of elderly (≥65 years) Medicare beneficiaries with type 2 diabetes mellitus who newly added rapid acting insulin to their oral antidiabetic drug regimen by rapid acting insulin persistence during one-year follow-up. Humana Medicare Advantage Prescription Drug Plan database 2007–2012 (row%).

	Persistence Measure 1					Persistence Measure 2				
	Persistent		Nonpersistent		*P* value	Persistent		Nonpersistent		*P* value
	*N*	Row%	*N*	Row%		*N*	Row%	*N*	Row%	
*All (N* = 2,334)	*429*	*18.4*	* 1,905 *	*81.6*		*787*	*33.7*	* 1,547 *	*66.3*	
*Number of OADs*					<0.001					<0.01
1 OAD	155	15.4	849	84.6		307	30.6	697	69.4	
2 OADs	187	19.1	790	80.9		336	34.4	641	65.6	
≥3 OADs	87	24.6	266	75.4		144	40.8	209	59.2	

Demographic characteristics										

*Gender*										<0.05
Female	228	18.9	978	81.1		432	35.8	774	64.2	
Male	201	17.8	927	82.2		355	31.5	773	68.5	
*Race*										
White	337	18.2	1,517	81.8		626	33.8	1,228	66.2	
African American	57	18.2	256	81.8		107	34.2	206	65.8	
Others	35	21.0	132	79.0		54	32.3	113	67.7	
Missing										
*Age in years*										
65–69 years	337	18.2	1,517	81.8		626	33.8	1,228	66.2	
70–74 years	57	18.2	256	81.8		107	34.2	206	65.8	
75–79 years	21	20.8	80	79.2		30	29.7	71	70.3	
80+ years	14	21.2	52	78.8		24	36.4	42	63.6	
*Region*										<0.001
Midwest	160	20.8	608	79.2		294	38.3	474	61.7	
South	221	16.9	1,086	83.1		396	30.3	911	69.7	
Northeast/west/others	48	18.5	211	81.5		97	37.5	162	62.5	

Health insurance characteristics										

*Type of plan*										
PFFS	171	20.6	661	79.4		302	36.3	530	63.7	
HMO	143	16.4	728	83.6		275	31.6	596	68.4	
PPO/others	115	18.2	516	81.8		210	33.3	421	66.7	

Baseline clinical characteristics										

*Any hypoglycemia*										
Yes	50	18.7	218	81.3		92	34.3	176	65.7	
No	379	18.3	1,687	81.7		695	33.6	1,371	66.4	
*Severe hypoglycemia*										
Yes	41	19.9	165	80.1		70	34.0	136	66.0	
No	388	18.2	1,740	81.8		717	33.7	1,411	66.3	
*aDCSI score*										
0	105	17.5	495	82.5		198	33.0	402	67.0	
1-2	143	19.9	574	80.1		256	35.7	461	64.3	
3-4	58	16.4	296	83.6		119	33.6	235	66.4	
5–13	123	18.6	540	81.4		214	32.3	449	67.7	
*A1C*										
<8%	62	16.7	310	83.3		120	32.3	252	67.7	
8-9%	23	14.1	140	85.9		47	28.8	116	71.2	
>9%	43	17.2	207	82.8		86	34.4	164	65.6	
Not available	301	19.4	1,248	80.6		534	34.5	1,015	65.5	

Baseline healthcare utilization										

*Any inpatient visit*					<0.05					
Yes	282	19.7	1,146	80.3		488	34.2	940	65.8	
No	147	16.2	759	83.8		299	33.0	607	67.0	
*Any ER visit*					<0.05					
Yes	263	20.0	1,055	80.0		456	34.6	862	65.4	
No	166	16.3	850	83.7		331	32.6	685	67.4	

Special conditions for the elderly										

*Polypharmacy*										
Yes (>14)	50	16.2	259	83.8		90	29.1	219	70.9	
No (≤14)	379	18.7	1,646	81.3		697	34.4	1,328	65.6	
*Cognitive impairment*					<0.001					<0.001
Yes	116	25.6	337	74.4		195	43.0	258	57.0	
No	313	16.6	1,568	83.4		592	31.5	1,289	68.5	
*Depression*										
Yes	86	21.7	311	78.3		139	35.0	258	65.0	
No	343	17.7	1,594	82.3		648	33.5	1,289	66.5	
*Fall*										<0.05
Yes	34	24.3	106	75.7		60	42.9	80	57.1	
No	395	18.0	1,799	82.0		727	33.1	1,467	66.9	
*Urinary incontinence*										
Yes	26	23.6	84	76.4		42	38.2	68	61.8	
No	403	18.1	1,821	81.9		745	33.5	1,479	66.5	

One-year follow-up basal insulin use										

*Basal insulin*					<0.05					<0.01
Yes	280	19.7	1,140	80.3		510	35.9	910	64.1	
No	149	16.3	765	83.7		277	30.3	637	69.7	

One-year follow-up RAI out-of-pocket cost per RAI prescription										

*Average OOP cost *					<0.001					<0.001
$0.0–<$6.3	112	22.0	398	78.0		180	35.3	330	64.7	
$6.3–<$36	38	16.0	200	84.0		85	35.7	153	64.3	
$36–<$56	62	8.9	636	91.1		106	15.2	592	84.8	
≥$56	217	24.4	671	75.6		416	46.8	472	53.2	

One-year follow-up out-of-pocket RAI cost per 30-day supply										

*OOP cost per 30-day supply*					<0.001					<0.001
$0.0–<$6.4	171	26.3	480	73.7		281	43.2	370	56.8	
$6.4–<$35.5	71	13.5	453	86.5		145	27.7	379	72.3	
$35.5–<$47.6	49	9.2	482	90.8		115	21.7	416	78.3	
≥$47.6	138	22.0	490	78.0		246	39.2	382	60.8	

Note: based on 2,334 elderly Medicare beneficiaries aged 65 years and above with type 2 diabetes mellitus, who were continuously enrolled in the Humana Medicare prescription drug plans for 18 months between 2007 and 2011 and added rapid acting insulin to their oral antidiabetic drugs regimen. These individuals had at least 2 claims for rapid acting insulin during the follow-up period. Significant group differences in persistence were tested with chi-square tests.

A1C: glycated hemoglobin; aDCSI: adapted-Diabetes Complications Severity Index; ER: emergency room; HMO: health maintenance organization; OAD: oral antidiabetic drug; PFFS: private fee-for-service; PPO: preferred provider organization; RAI: rapid acting insulin.

**Table 5:** Adjusted odds ratios and 95% confidence intervals from logistic regressions of rapid acting insulin persistence during 12-month follow-up among elderly (≥65 years) Medicare beneficiaries with type 2 diabetes mellitus who added rapid acting insulin to their oral antidiabetic regimen. Humana Medicare Advantage Prescription Drug Plan database 2007–2012.

	Persistence Measure 1			Persistence Measure 2		
	AOR	95% CI	*P* value	AOR	95% CI	*P* value
*Number of OADs*						
1 OAD	0.52	[0.38, 0.71]	<0.001	0.63	[0.48, 0.81]	<0.001
2 OADs	0.69	[0.51, 0.93]	<0.05	0.75	[0.58, 0.98]	<0.05
≥3 OADs						

Demographic characteristics						

*Gender*						
Female	0.97	[0.77, 1.21]		1.13	[0.95, 1.36]	
Male						
*Race*						
White						
African American	0.93	[0.67, 1.30]		0.98	[0.75, 1.29]	
Others	1.19	[0.71, 1.99]		0.78	[0.49, 1.23]	
Missing	0.96	[0.51, 1.79]		0.94	[0.55, 1.60]	
*Age in years*						
65–69 years						
70–74 years	0.91	[0.70, 1.20]		1.01	[0.81, 1.26]	
75–79 years	0.94	[0.69, 1.29]		0.96	[0.74, 1.25]	
80+ years	1.02	[0.73, 1.44]		1.20	[0.90, 1.59]	
*Region*						
Midwest	1.14	[0.79, 1.66]		1.00	[0.74, 1.36]	
South	0.91	[0.63, 1.31]		0.70	[0.52, 0.94]	<0.05
Northeast/west/others						

Health insurance characteristics						

*Type of plan*						
PFFS						
HMO	0.80	[0.60, 1.06]		0.88	[0.70, 1.11]	
PPO/others	0.88	[0.67, 1.16]		0.88	[0.70, 1.11]	

Baseline clinical characteristics						

*Any hypoglycemia*						
Yes	0.93	[0.65, 1.31]		0.99	[0.75, 1.32]	
No						
*aDCSI score*						
0	1.10	[0.79, 1.55]		1.08	[0.82, 1.43]	
1-2	1.20	[0.89, 1.60]		1.19	[0.94, 1.52]	
3-4	0.80	[0.56, 1.14]		0.98	[0.74, 1.30]	
5–13						
*A1C*						
<8%						
8-9%	0.85	[0.50, 1.45]		0.84	[0.55, 1.27]	
>9%	1.02	[0.65, 1.60]		1.07	[0.75, 1.53]	
Not available	1.08	[0.77, 1.49]		0.98	[0.76, 1.28]	

Baseline healthcare utilization						

*Any inpatient visit*						
Yes	1.03	[0.76, 1.41]		0.89	[0.69, 1.14]	
No						
*Any ER visit*						
Yes	1.12	[0.84, 1.49]		1.00	[0.79, 1.26]	
No						

Special conditions for the elderly						

*Polypharmacy*						
Yes (>14)	0.71	[0.51, 1.01]		0.73	[0.55, 0.96]	<0.05
No (≤14)						
*Cognitive impairment*						
Yes	1.41	[1.05, 1.89]	<0.05	1.50	[1.16, 1.92]	<0.01
No						
*Depression*						
Yes	0.99	[0.74, 1.34]		0.84	[0.65, 1.08]	
No						
*Fall*						
Yes	1.01	[0.65, 1.57]		1.15	[0.79, 1.68]	
No						
*Urinary incontinence*						
Yes	1.12	[0.69, 1.81]		0.98	[0.64, 1.48]	
No						

One-year follow-up basal insulin use						

*Basal insulin*						
Yes	1.32	[1.05, 1.66]	<0.05	1.33	[1.10, 1.60]	<0.01
No						

One-year follow-up RAI out-of-pocket cost per RAI prescription						

*Average OOP cost per RAI prescription*						
$0.0–<$6.3						
$6.3–<$36	0.48	[0.35, 0.66]	<0.001	0.50	[0.38, 0.65]	<0.001
$36–<$56	0.44	[0.32, 0.61]	<0.001	0.42	[0.32, 0.56]	<0.001
≥$56	0.41	[0.30, 0.55]	<0.001	0.54	[0.42, 0.70]	<0.001

Note: based on 2,334 elderly Medicare beneficiaries aged 65 years and above with type 2 diabetes mellitus, who were continuously enrolled in the Humana Medicare prescription drug plans for 18 months between 2007 and 2011 and added rapid acting insulin to their oral antidiabetic drugs regimen.

A1C: glycated hemoglobin; aDCSI: adapted-Diabetes Complications Severity Index; AOR: adjusted odds ratio; CI: confidence interval; ER: emergency room; HMO: health maintenance organization; OAD: oral antidiabetic drug; PFFS: private fee-for-service; PPO: preferred provider organization; RAI: rapid acting insulin.
